# Management of multiple valve replacement operations for a young patient with hypereosinophilic syndrome and valvular heart disease: a case report

**DOI:** 10.1186/s44215-024-00176-0

**Published:** 2024-11-14

**Authors:** Yuichiro Kado, Meikun Kan-o, Tomoki Ushijima, Satoshi Fujita, Gen Shinohara, Satoshi Kimura, Hiromichi Sonoda, Yasuhisa Oishi, Yoshihisa Tanoue, Akira Shiose

**Affiliations:** Department of Cardiovascular Surgery, Kyushu University, 3-1-1 Maidashi, Higashi-ku, Fukuoka, 812-8582 Japan

**Keywords:** Hypereosinophilic syndrome, Valve replacement, Surgical aortic valve replacement, Reoperation, Management, Cyclosporin

## Abstract

**Background:**

Hypereosinophilic syndrome (HES) is characterized by the overproduction of eosinophils and manifests as valvular disease and thrombogenesis. Herein, we report our experience with a patient with HES requiring multiple reoperations for prosthetic heart valve replacement via median sternotomy.

**Case presentation:**

The patient was a 54-year-old man who had undergone four valve replacement operations via median sternotomy (three mitral valve replacements and one double valve replacement) because of valvular diseases complicated by HES since he was 26 years old. All the artificial valves were bioprosthetic to prevent thrombotic events. At presentation, he had developed structural deterioration of the artificial aortic valve with severe stenosis. His prosthetic mitral valve did not fulfil the criteria for intervention, as it exhibited only mild regurgitation and no stenosis. The explanted mitral prosthetic valve at the previous (fourth) surgery had exhibited eosinophilic infiltration, resulting in the introduction of cyclosporin for poorly controlled HES. We conducted re-aortic valve replacement via a fifth median sternotomy using a bioprosthetic valve, and no eosinophilic infiltration was observed in the explanted valve. The patient was discharged on postoperative day 15 without complications.

**Conclusions:**

Controlling eosinophil count during the pre- and postoperative course is vital in treating patients with HES after valve replacement surgery. A holistic management and therapeutic strategy, including prosthetic valve selection and medication for HES is required to improve outcomes of patients with HES and heart valve disease.

## Background

Idiopathic hypereosinophilic syndrome (HES) is a rare disease in which eosinophils are overproduced and infiltrate into systemic organs, such as those of the cardiovascular and nervous systems, the skin, and lungs [1]. The effects of HES on the cardiovascular system include thromboembolic disease, valvular disease, myocarditis, and endocardial fibrosis [1]. Valvular diseases necessitate valve replacement surgery. However, patients with HES who undergo heart valve replacement may experience complications. Biological valves may undergo rapid structural valve deterioration (SVD), and mechanical valves pose the risk of valve thrombosis that is recalcitrant to anticoagulation therapy [2]. As mechanical valve dysfunction may cause rapid hemodynamic failure, biological valves are generally recommended for patients with HES. On the other hand, multiple reoperations for SVD of bioprosthetic valves increase the surgical risk for young patients with HES. Therefore, we report on a case in which we performed a reoperation for aortic valve replacement (AVR) via median sternotomy in a patient with HES who was young when he underwent his first heart valve replacement. We also describe our strategy of multiple reoperations.

## Case presentation

**Fig. 1:**
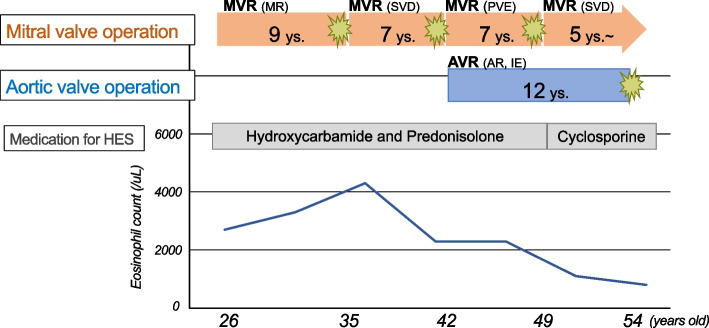
The patient’s clinical course. MVR mitral valve replacement, MR mitral valve regurgitation, SVD structural valve deterioration, PVE prosthetic valve endocarditis, AVR aortic valve replacement, AR aortic valve regurgitation, IE infective endocarditis, ys years, HES hypereosinophilic syndrome

**Fig. 2:**
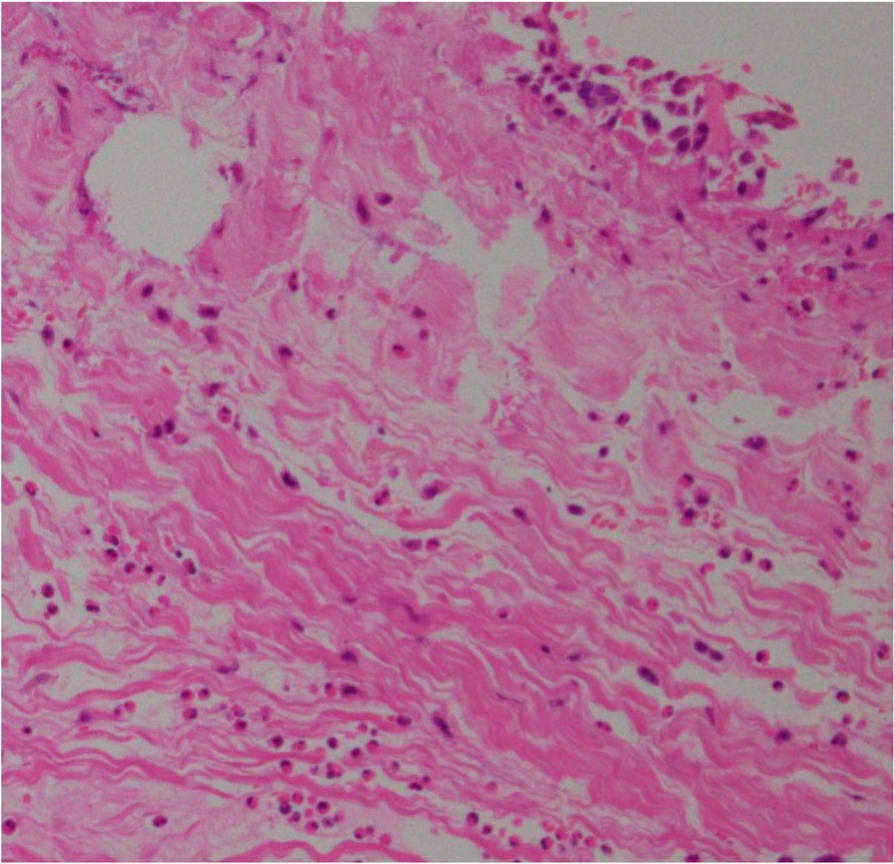
Pathological examination of the explanted mitral valve after the fourth operation. We observed eosinophilic infiltrates in the valve

**Fig. 3:**
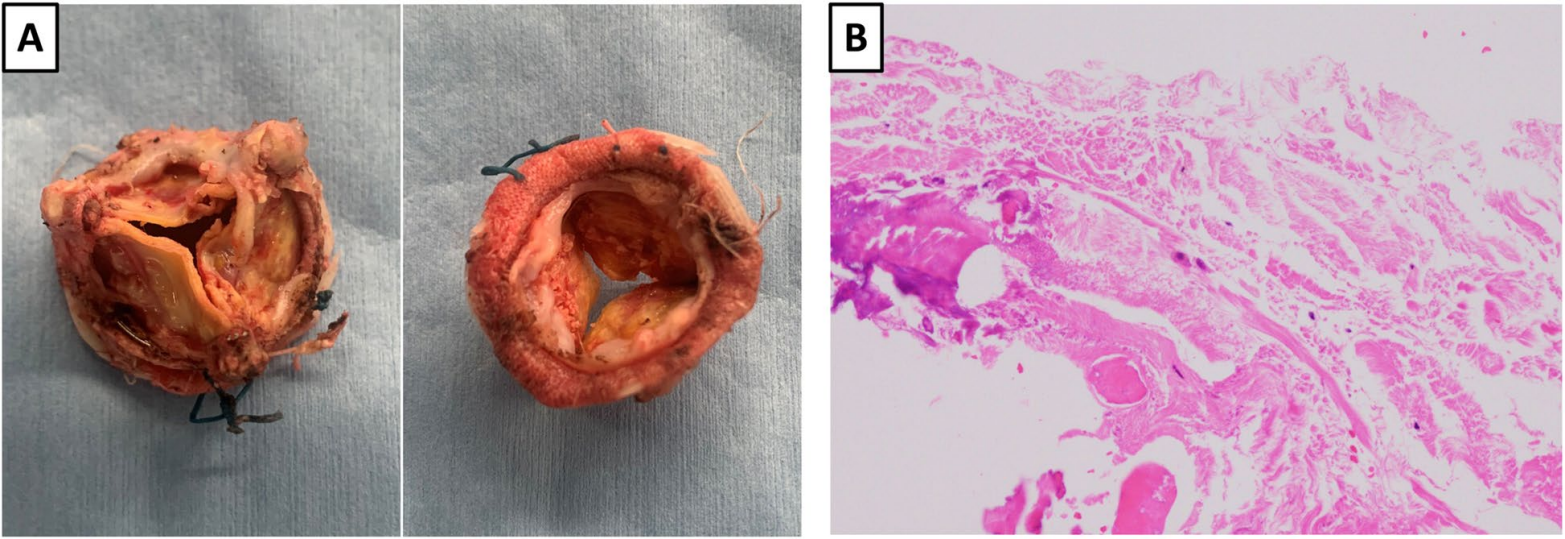
The explanted prosthetic aortic valve on the fifth operation. **a** The explanted valve exhibited thickened leaflets, calcification, and restriction of the opening. **b** Pathological examination revealed no eosinophilic infiltration in the explanted valve

A 54-year-old man (height, 172 cm; weight, 64 kg; body surface area, 1.75 m^2^) with HES, who was first diagnosed when he was 29 years old and underwent four median sternotomies for aortic and/or mitral valve replacement surgeries, was admitted for treatment for SVD of his prosthetic aortic valve. His operative history for HES was as follows: (1) At age 26, he underwent mitral valve replacement (MVR; Hancock 31-mm valve, Medtronic) for severe mitral valve regurgitation. (2) At age 35, he underwent re-MVR (CEP 31-mm valve, Edwards Lifesciences) for SVD. (3) At age 42, he underwent another re-MVR (CEP 29-mm valve) and an AVR (CEP Magna 21-mm valve, Edwards Lifesciences) for infective endocarditis of those valves. (4) Finally, at age 49, he underwent a third re-MVR (CEP Magna Mitral 29-mm valve) for SVD. The patient’s operative history, eosinophil count, and medications used for HES are displayed in Fig. 1. Pathological, microscopic examination of the explanted prosthetic mitral valve after the fourth operation revealed eosinophilic infiltration (Fig. 2). Accordingly, cyclosporin was introduced for HES because the HES status of the patient was judged to be poorly controlled. His main symptom at his admission to our hospital for his fifth median sternotomy was fatigability. Laboratory test revealed that the patient’s peripheral blood eosinophil count was 1100/µL (15% of the white blood cell count), and his brain natriuretic peptide concentration was slightly high (125 pg/mL). Echocardiography revealed thickening of the leaflets, calcification, and restriction of the opening of the prosthetic aortic valve, resulting in severe prosthetic aortic valve stenosis (aortic valve area, 0.5 cm^2^; peak velocity, 4.7 m/s). No valve stenosis and only mild regurgitation of the mitral prosthetic valve was observed. The patient’s left ventricular ejection fraction was normal (62%). Contrast-enhanced computed tomography revealed that the diameter of the prosthetic aortic valve was 21 mm. The patient underwent his fifth median sternotomy for re-AVR with a bioprosthetic valve (Inspiris RESILIA 21-mm valve, Edwards Lifesciences). The pathological examination revealed no eosinophilic infiltration of the extracted valve (Fig. 3). Cyclosporin was resumed in post operative day 2. Warfarin was administered as oral anticoagulation and targeted INR was 2.0 to 2.5. The patient was discharged on postoperative day 15 without complications.

## Discussion and conclusions

HES is a rare disease with an age-adjusted incidence rate of 0.036 per 100,000 [3]. All of the following criteria are necessary for a diagnosis of HES: (1) absolute eosinophil count > 1500 cells/µL for ≥ 1 month, (2) evidence of eosinophil-mediated organ damage, and (3) exclusion of all other potential causes of hypereosinophilia [4]. Lefebvre et al. reported that the overall survival of patients with HES was 80% at 5 years and 42% at 15 years [5], and the reported main cause of HES-related death is cardiac dysfunction [6]. Patients with HES can undergo surgery for valvular heart disease. However, heart valve replacement for HES remains challenging [7]. Mechanical valves can cause prosthetic valve dysfunction due to thrombosis despite the mandatory use of anticoagulation medication, whereas biological valves may also cause thrombosis or undergo rapid SVD. Bioprosthetic valves have yielded better surgical outcomes than mechanical valves [7]. Whereas, bioprosthetic valves have low durability, and young patients with HES require multiple operations owing to SVD, compounding the surgical risk. On the other hand, mitral valve repair is one of the effective procedures for HES patients with severe mitral valve regurgitation [8]; however, the mitral valve findings of the first operation of this patient were that the anterior leaflet was thickened at the edge and the commissure, and the posterior leaflet was thickened and shortened which showed no mobility at all. It might be caused by mitral valve tethering due to left ventricle enlargement. We judged that mitral repair was difficult; therefore, we decided to perform valve replacement in the first operation.

In this case, we replaced only mitral prosthesis valve because aortic prosthesis valve had no sign of SVD at the third re-MVR (Fig. 1). At that time, it had passed 7 years since the aortic prosthesis valve had been implanted; therefore, double valve replacement was another surgical plan because removing aortic prosthesis valve would provide a wide visual field for MVR. However, we could have comfortable visual field without removing aortic prosthesis valve; therefore, we just performed AVR.

Prosthetic valve selection is also one of the important operative strategies for HES patient. We selected a biological valve this time. One reason for this choice was the pathological features of the prosthetic mitral valve explanted following a previous operation when the patient was 49 years old. We deemed the patient to be at high risk of valve thrombosis if a mechanical valve was used, and concluded that a biological valve was a better choice. Quan et al. reported that they performed re-MVR with a newer type of mechanical prosthetic valve for a patient who had previously undergone bioprosthetic valve replacement for HES; no thrombotic events occurred in the next 4 years [9]. However, longer-term outcomes are required to evaluate these new types of mechanical prosthetic valves before their clinical use can be recommended.

**Fig. 4:**
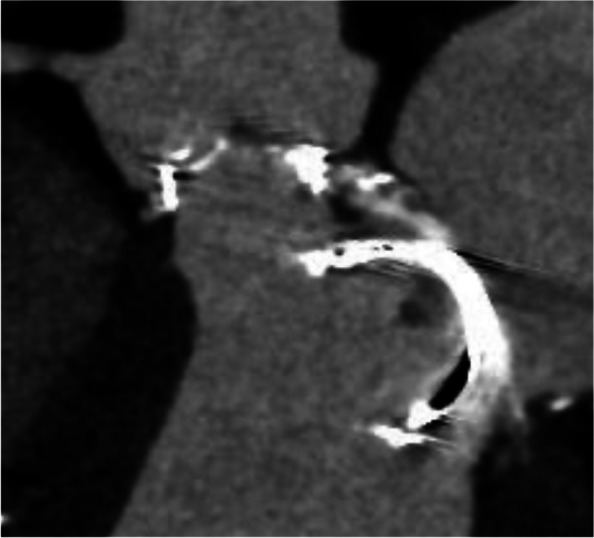
The prosthetic valve strut at the mitral position protrudes into the left ventricular outflow tract

Transcatheter aortic valve implantation (TAVI) is an excellent procedure for patients with a high surgical risk. Transcatheter aortic valve-in-surgical aortic valve (TAV-in-SAV) is an alternative to surgical reoperation for SVD. We considered TAV-in-SAV in this case but discovered that the mitral strut of the prosthetic mitral valve would interfere with ballooning when the balloon-expandable device would be used during TAVI (Fig. 4). Moreover, self-expandable devices pose a risk of adhesions, especially in patients with HES who require frequent reoperation for valve replacement. Therefore, we decided against TAV-in-SAV for this patient.

The key role of pre- and postoperative management of patients with HES is to control their eosinophil count. Aggressive treatment of HES is imperative to reduce the eosinophil count and minimize valvular damage [10]. In our case, the explanted prosthetic valve following the patient’s most recent (fourth) operation exhibited eosinophilic infiltration, whereas the explanted prosthetic valve following the fifth operation did not. This may be because of the aggressive use of cyclosporin following his fourth operation. Controlling the eosinophil count may reduce damage to the prosthetic valve and the number of reoperations for bioprosthetic valve replacement required by young patients with HES. We assume that the HES patients would need multiple valve operations, and to protect the surrounding tissue of the aortic and mitral annulus is important. When the mitral prosthetic valve would have some signs of SVD in the future, we may undergo single valve replacement on mitral valve, if the aortic valve would have no sign of SVD. However, when the mitral prosthetic valve would have some signs of SVD, we have to perform double valve replacement procedure if the aortic valve would have some signs of SVD. On the other hand, removing the pre-existing aortic prosthesis valve would provide better visual field at the time of MVR. We should plan double valve replacement depending on the visual field of the operation, or the passed years since the last AVR.

In conclusion, we performed the fifth reoperation for prosthetic heart valve replacement via median sternotomy for a patient with HES. Bioprosthetic valves are generally recommended for such patients; however, multiple surgeries are inevitable for young patients owing to SVD. A holistic management and therapeutic strategy, including prosthetic valve selection and medication for HES to control eosinophil count, as well as the development of new types of antithrombotic prosthetic valves are required to improve outcomes of patients with HES and valvular heart disease.

## Abbreviations

HES: Hypereosinophilic syndrome
SVD: Structural valve deterioration
MVR: Mitral valve replacement
AVR: Aortic valve replacement
TAVI: Transcatheter aortic valve implantation
TAV-in-SAV: Transcatheter aortic valve-in-surgical aortic valve
